# Systemic coagulation parameters in mice after treatment with vascular targeting agents

**DOI:** 10.1186/1477-9560-3-21

**Published:** 2005-12-10

**Authors:** Maike Unruh, Andrea Grunow, Claudia Gottstein

**Affiliations:** 1 Department of Internal Medicine I/Experimental Oncology and Vascular Biology, University Hospital Cologne, Joseph-Stelzmann-Straße, 50924 Cologne, Germany

## Abstract

**Background:**

Vascular targeting of malignant tumors has become a clinically validated new treatment approach with clear patient benefit. However clinical studies have also revealed that some types of vascular targeting agents (VTAs) are prone to coagulation system side effects. It is therefore essential to predetermine coagulation parameters in preclinical studies. As of to date, this has rarely been done, predominantly due to technical issues.

The goal of this study was to establish and apply a standardized process, whereby systemic coagulation activation can be routinely measured in mice.

**Results:**

We have evaluated a number of sampling techniques and coagulation tests regarding their suitability for this purpose. We were able to adapt two assays measuring soluble fibrin, a marker for a prethrombotic status. Thus, soluble fibrin could be measured for the first time in mice. All assays were validated in a positive control model for systemic coagulation activation, i.e. lipopolysaccharide-induced endotoxemia.

Based on our results, we selected a panel of coagulation tests, which are both feasable and informative for preclinical testing of VTAs: soluble fibrin, thrombin-antithrombin complexes, free antithrombin III, white blood cell counts and platelet counts. The effect of tumor transplants on coagulation parameters was evaluated using this panel. We then applied this set of assays in treatment studies with a VTA developed in our laboratory to investigate a potential systemic coagulation activation.

**Conclusion:**

We have established a standardized panel of assays that can be used to test murine blood samples for coagulation activation in preclinical studies. All tests are feasible to perform in any research laboratory without specialized equipment. In addition, this is the first report to measure soluble fibrin, an early marker of systemic coagulation activation, in mice. The panel was applied on tumor bearing mice and mice treated with a VTA. We suggest its general application for coagulation activation analyses in mice.

## Background

The tumor vasculature has been recognized as a promising target for new treatment approaches such as angiogenesis inhibitors, vascular disrupting agents and specific vascular occluding agents [1-5]. A number of these vascular targeting agents (VTAs) have been evaluated in clinical trials and prolongation of survival has been demonstrated in a phase III trial [6]. However in several studies serious side effects on the coagulation system, such as myocardial infarction, thrombembolic complications and fatal bleedings have been observed [7-11]. In addition, in cancer patients the underlying disease can also interact with the coagulation system [reviewed in [12]].

Therefore, assessment of coagulation parameters is of importance in preclinical trials studying the effect of VTAs as anti-cancer agents.

Surprisingly few data are available on coagulation parameters in preclinical VTA-treatment studies in mice. This seems to be predominantly due to technical challenges: Sample volumes in mice are small; artifacts are easily introduced; and the number of tests which are suitable for murine samples is limited because most commercially available coagulation assays designed for use in humans are not applicable in mice [13]. Extensive coagulation studies have therefore been restricted to larger animals, such as rabbits, rats and dogs [14-16]. However mice are the species most widely used for preclinical studies in experimental oncology.

Although there is a vast amount of literature on mouse models concerning thrombosis or lipopolysaccharide (LPS)-induced disseminated intravascular coagulation (DIC), in most studies only a few coagulation parameters were measured simultaneously. The lack of consistency in the selected parameters makes a comparison between different reports difficult [16-24]. Also technical issues such as sampling techniques and anticoagulant additives vary: In some experiments blood is sampled by *Vena cava *puncture [25], in others by heart puncture or by tail cut [26,27], and citrate:blood ratios of 1:10 or 1:5 have been reported.

In this study, we have set out to establish a standardized process for the measurement of potential side effects of drugs, that might affect the coagulation system. In addition to optimizing technical issues, we wanted to assemble a panel of tests, that would i) detect coagulation changes with a focus on systemic coagulation activation, ii) be suitable for murine samples, iii) be feasable to perform routinely in a large number of samples and iv) be preferentially available commercially. The tests evaluated here were: Prothrombin time (PT), a global coagulation marker indicative of changes in the extrinsic and the common pathway; activated partial thromboplastin time (aPTT), a global marker indicative of changes in the intrinsic and common pathway; soluble fibrin (SF), a sensitive marker of systemic coagulation activation; thrombin-antithrombin complexes (TAT), a marker of systemic coagulation activation; functional antithrombin III (ATIII), which is reduced in consumption coagulopathy; white blood cell counts (WBC), which show a specific pattern of change over time in DIC [28]; platelet counts (PLT), which are typically reduced in DIC; and schistocyte counts, i.e. fragmented erythrocytes, which can also be indicative of DIC.

Soluble fibrin (SF) had not been measured in mice, although it was found to be a useful marker for a prethrombotic state in humans [29-31]. SF occurs after thrombin cleavage of fibrinogen. It is not a clearly defined homogenous molecule but rather describes various fibrin compounds of different molecular structures, including fibrin monomers, polymers and crosslinked fibrin. In the past, a number of assays detecting soluble fibrin were developed [reviewed in [32]]: Gelation tests with ethanol or protamine [33,34], chromatographic analysis [35], Western Blotting of fibrin related compounds [35,36], immunological methods such as ELISAs or latex agglutination assays [37] and functional assays. The former are neither very sensitive nor suited for routine analysis, while the immunological methods are species specific and do not crossreact with murine samples (13, own data). Among the functional tests, two different principles have been exploited for test development: i) the agglutination reaction of fibrin-coated erythrocytes with SF in plasma samples [38], and ii) the ability of SF to accelerate the tPA (tissue plasminogen activator)-catalyzed cleavage of plasminogen to plasmin [39]. These two tests were developed for human samples and made commercially available. Initially they were not suited for testing murine samples, therefore we modified them for the use in mice.

We then selected a panel of assays suited for testing systemic coagulation assays in mice. This test series was applied in mouse models to investigate the impact of tumor transplants on the coagulation system, and to predetermine potential side effects caused by VTAs.

## Results

### Blood sampling from the tail vein or heart introduces artifacts

Three different sampling techniques were compared in untreated control mice: tail cut, heart puncture and *Vena cava *puncture. Tables 1 and 2 summarize the results.

**Table 1:** Comparison of sampling techniques for different coagulation parameters: Vena cava puncture (VCP) vs tail cut (TC) as measured by Prothrombin Time (PT) and activated Partial Thromboplastin Time (aPTT) in plasma of untreated negative control mice

Assay:	PT mean (SD)		aPTT mean (SD)	number of samples not evaluable	number of samples analyzed	% of samples not evaluable
Sampling technique:	[s]	[% of standard]*	[s]	[n]	[n]	[%]

						
VCP 1:5	9.2 (1.0)	94 (10)	31.7 (4.6)	3	15	20%
VCP 1:10	7.8 (0.1)	116 (13)	23.2 (0.8)	0	10	0%
TC 1:5	-	-	-	3	3	100 %
TC 1:10	-	-	-	4	4	100 %
						
Ref.values:	8–10	80–120	32–36/23–25†			

* where the clotting time of a normal plasma standard was defined as 100%

† normal values depended on citrate:blood ratio, and were 32–36 s for 1:5 ratio and 23–25 s for 1:10 ratio

**Table 2:** Comparison of sampling techniques for different coagulation parameters: Vena cava puncture (VCP) vs heart puncture (HP) as measured by Soluble Fibrin in plasma of untreated negative control mice

	Number of mice negative	Number of mice with borderline result	Number of mice positive	Number of mice analyzed	Number of positive results in negative control mice
sampling technique:	n	n	n	n	%

					
VCP 1:5	17	1	0	18	0 %
VCP 1:10	10	2	5	17	29%
HP 1:5	0	0	5	5	100%
HP 1:10	3	0	10	13	77%
					
Ref.values:	neg‡				

‡ negative, if no further manipulations are performed. If reagents are administered i.v., there can be up to 30% positive samples, independent of the reagent applied (see Fig. 2).

Table 1 shows a comparison of results from measurements of PT and aPTT in samples obtained either by *Vena cava *puncture or by tail cut. Citrate dilutions of 1:5 and 1:10 were used as indicated. None of the samples obtained by tail cut were evaluable because they coagulated prior to the test. As expected, the clotting times for 1:5 and 1:10 citrate dilutions differed slightly. The samples diluted with citrate at a 1:5 citrate:blood ratio showed a high standard deviation, and some samples were not evaluable at all. This was most likely due to a relative lack of Ca^++ ^ions. Adjusting the Ca++ ion concentration might have circumvented this problem. However, due to the high sample volume required for PT and PTT measurements, plasma samples could not be analyzed simultaneously for PT/PTT and for the panel of SF, ATIII, TAT, WBC and PLT. Therefore it was not necessary to use the 1:5 citrate:blood ratio, when analyzing PT and PTT. Similarly, when we analyzed samples for thrombin-antithrombin complexes or soluble fibrin, samples obtained by tail cut were either not evaluable or showed systemic coagulation activation (data not shown). In conclusion, tail cut seems to be an unsuitable blood sampling method for coagulation parameter analysis.

In Table 2, a comparison of results from soluble fibrin (SF) determinations in *Vena cava *puncture samples and heart puncture samples at different citrate:blood ratios are shown. Only samples obtained by *Vena cava *puncture at a 1:5 citrate:blood ratio were negative for SF, as expected in untreated mice. Since assays for TAT, WBC and PLT could also be successfully performed using this sampling technique (data not shown), *Vena cava *puncture at a citrate:blood dilution factor of 1:5 was our preferred sampling method (with the possible exception of PT and aPTT).

### Measurement of SF in mice

**Figure 1:**
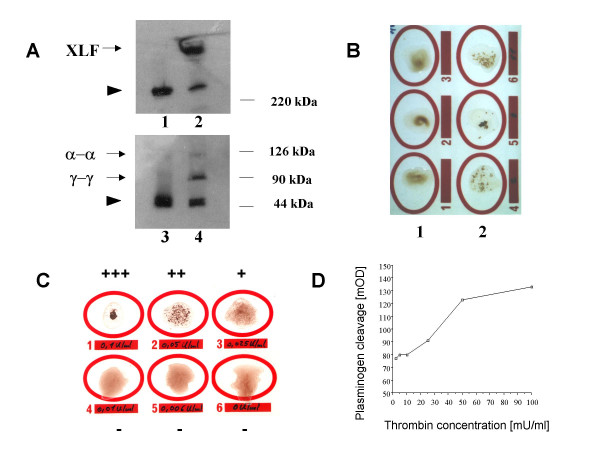
***In vitro *generation and measurement of murine soluble fibrin. **A. Western Blotting. Murine plasma samples were treated with 0.02 U/ml thrombin and separated via gel electrophoresis under non-reducing (upper panel) and reducing (lower panel) conditions. Fibrin(ogen) was detected with a polyclonal anti-fibrinogen antibody. Lanes 1, 3: plasma without thrombin treatment. Lanes 2, 4: thrombin-treated plasma. Lane 1: Arrowhead points at undigested 340 kDa fibrinogen hexamer (αβγ)_2_. Lane 2: upper band depicts high molecular weight cross linked fibrin (XLF), lower band represents remaining undigested fibrinogen. Lane 3: Arrowhead points at fibrinogen monomers consisting of α- (64 kDa, not visible), β-(56 kDa) and γ- (47 kDa) chains. Lane 4: formation of α-chain dimers (α-α, weakly stained) and γ-chain dimers (γ-γ); lower band represents fibrinogen β- chains and remaining non cross-linked γ-chains. B. Agglutination test for soluble fibrin. The same samples as in A were evaluated with the SF agglutination test as described in the Methods section. 1: Untreated plasma samples show no agglutination. 2: Thrombin-treated plasmas agglutinated and gave a positive test result. C. Semiquantitative measurement of soluble fibrin. Murine plasma samples were treated with different amounts of thrombin (100 mU/ml, 50 mU/ml, 25 mU/ml, 10 mU/ml, 6 mU/ml and 0 mU/ml as indicated). For a direct comparison with the chromogenic assay shown in panel D, and in contrast to the regular agglutination test protocol, thrombin was added before dilution of the sample in Owren's buffer followed by incubation at 37°C for 10 min. The amount of thrombin used for activation of the plasma correlated with the agglutination reaction, thus allowing for semiquantitative evaluation of test results. D. Quantitative analysis of soluble fibrin by a chromogenic assay. Murine plasma samples were treated with different amounts of thrombin and absorption at 405 nm was correlated with the thrombin concentration used to induce soluble fibrin. There was a linear relationship beween 15 mU/ml and 50 mU/ml thrombin.

First we induced SF *in vitro *by treatment of murine or human plasma with thrombin, and verified that SF formation had taken place using Western Blotting (Figure 1A).

Two functional assays developed to measure SF in human samples were then adapted for the use in mice:

The erythrocyte agglutination assay is an easily performed test kit, which measures SF in a semiquantitative fashion. When we carried it out according to the manufacturers instructions, but using murine samples, we observed agglutination reactions in our negative control samples (normal mouse plasma). This was most likely due to an unspecific bridging factor. However, after varying assay conditions (dilution of plasma, reagent volumes and reagent conditions) we were able to eliminate the unspecific agglutination. Using the optimized protocol as described in the methods part, the positive controls, i.e. thrombin-treated samples, which had been shown by Western Blotting to contain SF, showed an agglutination reaction, which was not observed in negative control samples (untreated normal mouse plasma). This is demonstrated in Figure 1B. We then treated plasma samples with various doses of thrombin, resulting in corresponding amounts of SF. Figure 1C illustrates that the agglutination reaction was visibly stronger in the presence of higher amounts SF, yielding a semiquantitative evaluation.

The chromogenic assay is more difficult and time consuming. It originally was developed for automated measurements, but we established a manual procedure which measured 12 samples per test. Positive controls for the standard curve and negative controls were generated as described in the Methods section. The standard curve was linear for normal mouse plasma standards treated with 15 mU/ml to 50 mU/ml thrombin. Thus, within this range, sample values could be quantitated in arbitrary units corresponding to the amount of activating thrombin (Figure 1D). A direct conversion into μg/ml SF was not possible, because human plasma reacted differently than mouse plasma (data not shown), and therefore the human standards provided in the kit could not be used in the murine setting.

The detection limits were comparable for the agglutination assay and the chromogenic assay (Figure 1C, D). Positive controls of the chromogenic assay test kit were also positive in the agglutination test. When both tests were performed with the same plasma samples, their results correlated with each other and with a third test, the TAT assay (Table 3): In mice injected with 20 μg LPS i.p., 4/5 mice became positive in all three assays. When 300 U of hirudin was injected in addition to 20 μg LPS, all but one mouse in the Berichrom FM test, were negative for SF or TAT in all three assays. Thus we confirmed the thrombin specificity of the signals observed in the three assays.

**Table 3:** Correlation of results of three different assays determining thrombin activity in plasma of mice treated with lipopolysaccharides (LPS) in the presence and absence of the thrombin inhibitor hirudin

		Soluble Fibrin determined by agglutination assay	Soluble Fibrin determined by chromogenic assay	Thrombin-Antithrombin complexes	
Treatment of mice	sample no.	semiquantitative result*	qualitative result (fold increase over negative control)	qualitative result	[ng/ml]

					
LPS, 15 min	1	-	- (1.1)	-	3
	2	-	- (1.3)	-	0
	3	-	- (1.1)	-	2
	4	(+)	+ (2.2)	-	3
	5	++	+ (3.7)	-	8
LPS, 2 h	6	(+)	- (1.2)	-	6
	7	+	+ (2.2)	+	21
	8	+	+ (2.2)	+	34
	9	++	+ (2.4)	+	20
	10	++	+ (2.1)	+	22
LPS + hirudin, 2 h	11	-	+ (2.2)	-	10
	12	-	- (1.6)	-	10
	13	-	- (1.5)	-	10
	14	(+)	- (1.8)	-	5
	15	(+)	- (1.8)	-	5
no treatment	16	-	- (1.5)	-	2
	17	-	- (1.1)	-	0
	18	-	- (0.9)	-	0

* where ++ corresponds to 0.05 thrombin units/ml, + corresponds to 0.025 thrombin units/ml, (+) stands for borderline result (less than +, but not conclusively -) and – stands for negative (see also Figure 1C).

SF was also measured in another model of systemic coagulation activation: 2 U of thrombin were injected i.v. into mice, and 7 out of 9 mice showed positive test results. The remaining two mice probably failed to develop a systemic coagulation activation because thrombin initiated a strong local coagulation reaction in the tail vein, consuming the vast majority of thrombin at the injection site.

### I.v. injections of an inert solution can activate the coagulation system

**Figure 2:**
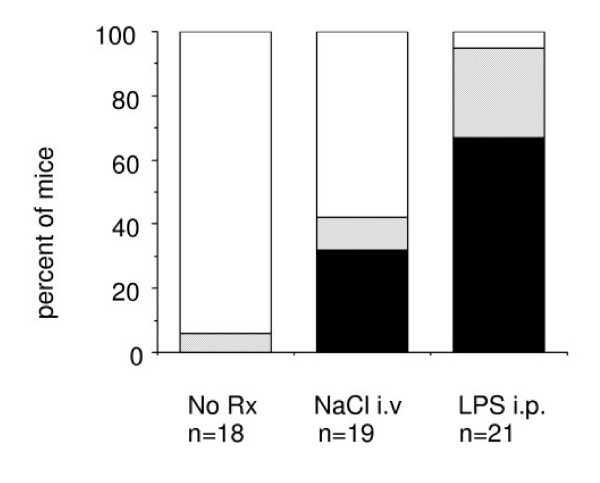
**Occurrence of soluble fibrin (SF) in mice injected i.v. with 0.9% NaCl. **The percent of mice, that became positive for soluble fibrin in the SF agglutination test after slow i.v. injection of physiological NaCl-solution (clinical grade), is plotted *versus *non-injected mice ("No Rx") and *versus *Lipopolysaccharide (LPS)-injected mice as a positive control. Black: percent positive, shaded: percent borderline, white: percent negative for SF.

Comparisons between untreated mice and mice treated with 0.9% NaCl-solution (clinical grade) revealed that i.v. injections can induce the formation of SF. Figure 2 demonstrates that SF became detectable in 32 % of plasma samples from mice after a slow i.v. injection of 0.4 ml physiological NaCl-solution in comparison with 0% in untreated mice. LPS-treated mice served as a positive control. This demonstrates that i.v. injection of an inert solution unrelated to the coagulation system can result in systemic coagulation activation, likely by mechanically damaging the venous wall. The agglutination test for SF was sensitive enough to detect this reaction.

### All test parameters become positive in a mouse model of systemic coagulation induction

**Figure 3:**
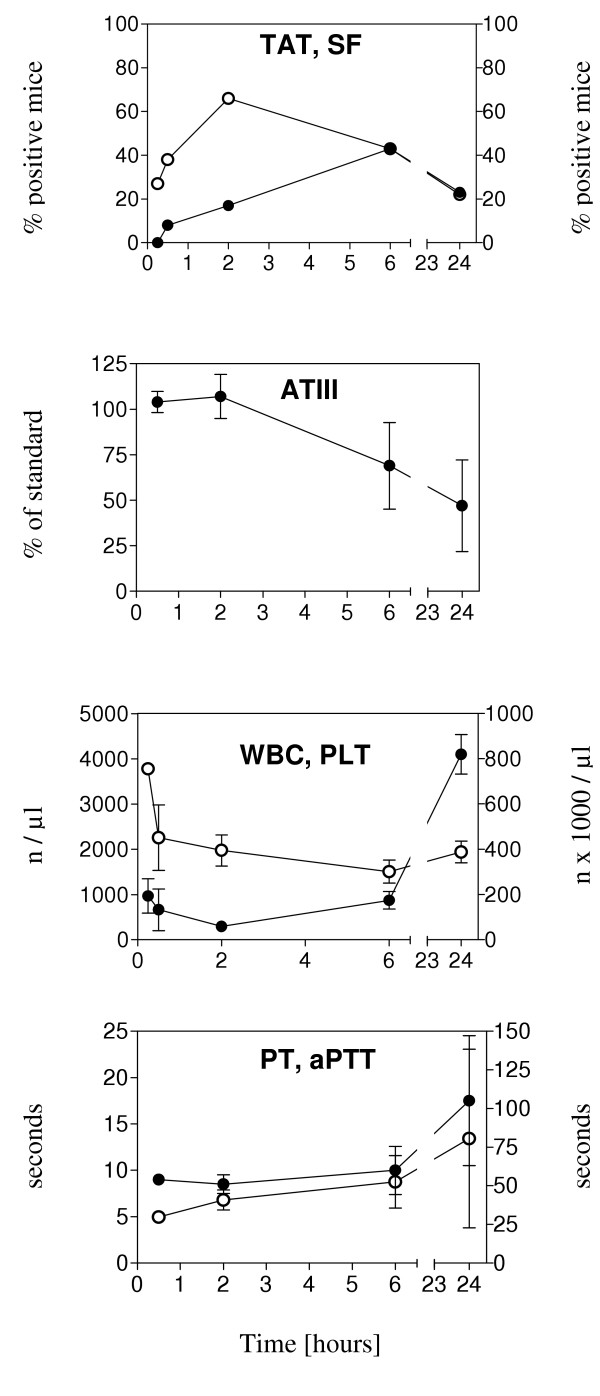
**Changes of coagulation parameters in Lipopolysaccharide-induced endotoxemia. **Mice were injected with 50 μg Lipopolysaccharide (LPS) i.p. and coagulation parameters were analyzed as described in the text. Black circles, left y-axis: TAT, ATIII, WBC, PT; White circles, right y-axis: SF, PLT, aPTT. Means and standard deviations (error bars) from groups of 3–15 mice are shown.

As illustrated in Figure 3, all test parameters changed over time, after injection of LPS. SF became positive at early time points, TAT and free ATIII at later time points (6 h and 24 h after LPS injection). The counts for WBC and PLT showed a pattern, which was previously described for humans [28], i.e. WBC counts fell initially, followed by an increase above normal, and PLT counts were reduced. PT rose after 24 hours, but not in all mice, therefore standard deviations were high. aPTT was elevated as early as 2 h post injection and continued to rise. Schistocyte counts changed only slightly (from 3 to 6 fragments per field after LPS injection and from 4 to 9 fragments per field after tumor necrosis factor-alpha injection, data not shown). In conclusion, all parameters tested here are suited for detecting a systemic coagulation activation, with schistocyte counts being the least sensitive.

### WBC and PLT counts can be elevated in tumor bearing mice

**Figure 4:**
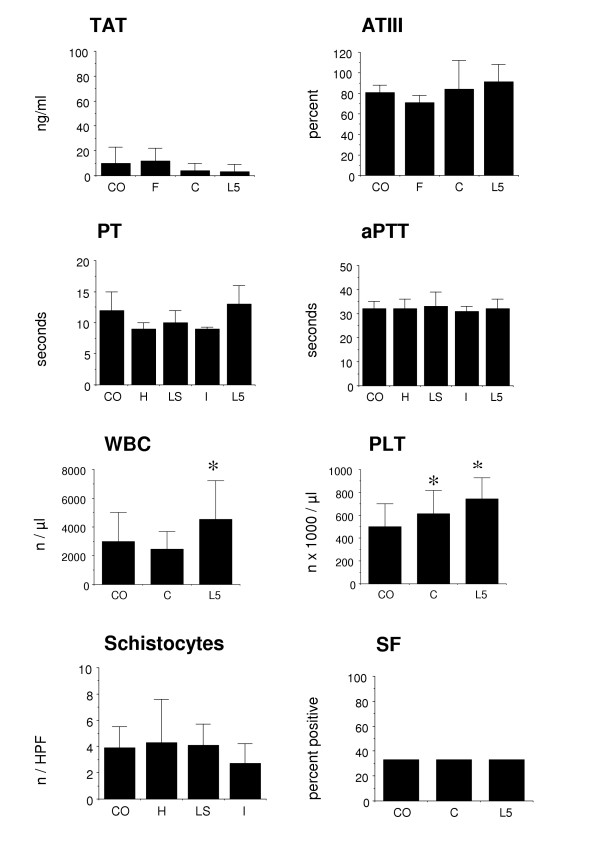
**Changes of coagulation parameters in tumor bearing mice. **Tumor bearing mice were analyzed for coagulation parameters as described in the text in comparison with non-tumor bearing mice. CO: non-tumor bearing control mice; F: mice with F9 teratocarcinoma; C: mice with Colo677 myeloma; L5: mice with L540rec Hodgkin's lymphoma; H: mice with HT29 colon carcinoma; LS: mice with LS174T colon carcinoma; I: mice with IMR5 neuroblastoma. Means and standard deviations (error bars) from groups of 4–18 mice are shown. *: statistically significant *versus *control mice (p < 0.05).

We measured each of the laboratory parameters in several subcutaneously growing tumor models and compared the values to those of non-tumor bearing mice (Figure 4). In the models tested, we did not find any significant changes in laboratory parameters compared to control mice, except for WBC and PLT. WBC counts were elevated in the lymphoma model and PLT counts both in the myeloma and the lymphoma model.

This argues against a procoagulant status in these models, but would have to be evaluated separately for other tumor models.

### Selection of a panel of coagulation parameters for routine analysis

The selection of tests was based on the following criteria: Sensitivity of the test, suitability for measuring systemic coagulation activation, sample volume required and the feasability for testing a large number of samples. The required sample volume is even more critical in studies with diseased mice, because in our experience the volume of blood that can be obtained from sick mice is lower than that from healthy mice.

In consequence, the measurement of PT and aPTT was not included in the panel because i) a total of at least 100 μl plasma (about 200 μl of blood) is needed for these two tests; ii) it is well accepted that PT and aPTT are global markers and not specific for systemic coagulation activation [40]; and iii) a comparison of aPTT results between different laboratories is difficult [41].

Schistocyte counts are very labour intensive, somewhat subjective, and did not prove to be very sensitive. Therefore they were not included in the panel.

In contrast, SF and TAT indicate a systemic coagulation activation and the required sample volumes are low (5 μl and 20 μl of plasma, respectively). ATIII measures a different parameter (consumption) with a different kinetics and also requires a low sample volume (5 μl of plasma). WBC and PLT, which can both be performed with a total sample volume of 10 μl blood, have a known pattern of change in DIC.

In conclusion, we propose SF, TAT, ATIII, WBC and PLT as a useful panel of tests for systemic coagulation activation.

### A VTA consisting of the extracellular domain of tissue factor targeted to VCAM-1 does not induce systemic coagulation activation

**Figure 5:**
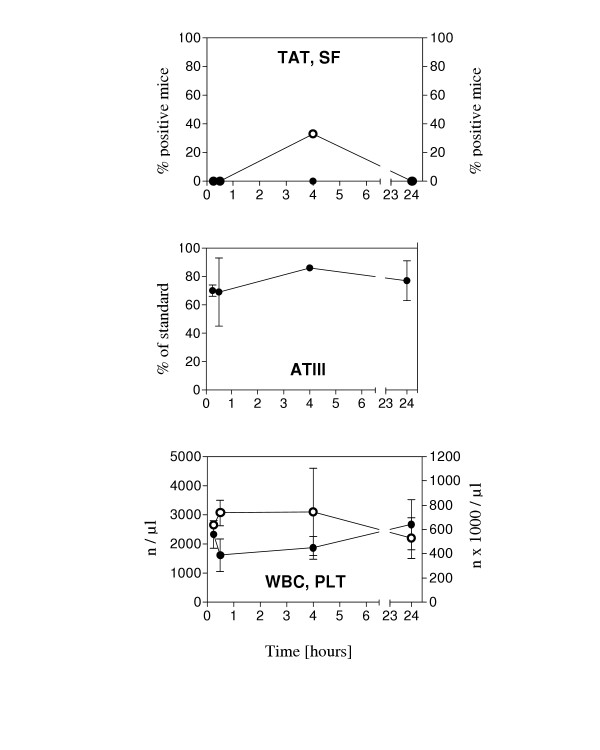
**Changes of coagulation parameters after treatment with vascular targeting agent. **Mice with Colo677 myeloma tumors were injected i.v. with a vascular targeting agent, consisting of the extracellular portion of tissue factor targeted to murine VCAM-1. Coagulation parameters were analyzed as described in the text. Black circles, left y-axis: TAT, ATIII, WBC. White circles, right y-axis: SF, PLT. Means and standard deviations (error bars) from groups of 3–6 mice are shown.

We then tested the impact of a VTA, which selectively induces coagulation activation in tumor vasculature [5], on coagulation parameters at different time points after injection into tumor bearing mice. The results are illustrated in Figure 5 and demonstrate that there was no indication of systemic cogulation activation after treatment with that particular VTA.

## Discussion

Despite the importance of measuring coagulation parameters in preclinical treatment studies with VTAs, there have been virtually no reports on this topic. This is predominantly due to technical challenges. Therefore our first task was to select and refine tests for detection of systemic coagulation activation in mice. Regarding technical issues, we observed that sampling from the heart or tail vein introduced artifacts in coagulation measurements. The direct comparison of three different sampling techniques demonstrated that tail cut and heart puncture appear to be unsuitable for coagulation studies. This is due to tissue damage, which results in artificial coagulation activation leading to positive test results for early markers of coagulation activation. Therefore, we propose *Vena cava *puncture as the sampling method of choice. A second potential source of coagulation activation is the application route. Our observation that SF formation occurred after the slow i.v. injection of an inert solution, is important since i.v. injections are frequently used in preclinical studies. This must be considered for the selection of appropriate negative controls and reference values.

Systemic coagulation activation can ultimately lead to disseminated intravascular coagulation (DIC), a complex thrombohemorrhagic disorder. Diagnosis of DIC is complicated because there is no truly specific laboratory test available. Various coagulation tests have been ranked in terms of their reliability to detect DIC in the following order [40]: 1. Prothrombin fragments 1 and 2, 2. Fibrin D-dimer, 3. Antithrombin III, 4. Fibrinopeptide A, 5. Platelet factor 4, 6. Fibrin degradation products, 7. Platelet counts, 8. Protamine test detecting SF, 9. thrombin time, 10. fibrinogen, 11. PT, 12. aPTT. Since the commercially available assays for prothrombin fragments 1 and 2, fibrin D-dimer, fibrinopeptide A, platelet factor 4 and fibrin degradation products are not suited for the analysis of murine samples (13, own data), we tested antithrombin III, TAT, platelet counts and soluble fibrin. Soluble fibrin is diagnostic for thrombin action on fibrinogen and has been described as an early marker for systemic coagulation activation [29-31]. This is the first study to describe the measurement of SF in mice. We have adapted two commercially available functional SF assays for this purpose. They correlated well with each other and with TAT levels in a mouse model for DIC. Hirudin, a direct thrombin inhibitor, abolished the response indicating that the results were due to a specific action of thrombin. We did not include the measurement of fibrinogen, because it has been described to be neither sensitive nor specific for systemic coagulation activation [42]. Since our proposed sampling technique requires that for every time point one mouse be sacrificed, it becomes important, that all tests be performed with a reasonable sample volume. Tests with different kinetics should be combined to increase the probability for detection of systemic coagulation activation at any given timepoint. Tests with low specificity for systemic coagulation activation or tests which require high sample volumes are of lesser value, e.g. PT and aPTT.

Based on the criteria of feasability and suitability we selected the following panel of assays to detect systemic coagulation activation: SF, TAT, ATIII, WBC and PLT. The complete set of selected tests can be performed with 50 μl of plasma, the tests are suited for testing in a large number of samples, they all become positive in a mouse model of DIC, and they are generally available.

We then analyzed the impact of tumors on the murine coagulation status. In the tumor models used here there were no significant changes in most of the coagulation parameters, but this remains to be validated separately for each tumor model used.

We have since applied this panel routinely in preclinical studies with VTAs developed in our laboratory, and one example is presented. For that particular VTA, all coagulation parameters stayed within normal range over the entire time period tested.

## Conclusion

Vascular targeting is a new and promising approach for the therapy of cancer and other diseases. Clinical studies have revealed, that there is a potential for side effects in the coagulation system. Therefore, it is essential to predetermine these potential side effects in preclinical studies. Testing of coagulation parameters in mice, the most frequently used species for these trials, has so far been reserved to specialized laboratories. In this study a panel of assays and optimized methods for measuring systemic coagulation activation in murine samples is presented. The panel was applied on tumor bearing mice and on mice treated with a VTA. We suggest its general use for coagulation activation analysis in mice.

## Methods

### Blood sampling

Three different techniques were used: *Vena cava *puncture, heart puncture and tail cutting. If not otherwise specified, blood was drawn from the *Vena cava.*

For *Vena cava *or heart puncture, mice were anaesthetized with ether, the thoracical cavity was opened, and blood was drawn from the upper *Vena cava *or from the heart into a syringe containing sodium citrate (1/10 or 1/5 volume of 3.1% sodium citrate). For tail cutting, mice were warmed up under an infrared lamp (250 W) for one minute. The tail was cut with a scalpel, the first three drops of blood were not used, and then blood was collected into a heparin-coated centrifuge tube, which contained sodium citrate (see above). After removing blood for blood cell counts (WBC, PLT, schistocytes), citrated blood was centrifuged twice (500 g and 8000 g) to remove platelets. Platelet-poor plasma was analyzed immediately for soluble fibrin and the remainder of the samples were frozen at -80°C until further use.

### Negative control plasma

Negative control mice were either untreated mice or mice injected with 0.9% NaCl-solution (clinical grade). In some experiments pooled plasma was used, which was prepared as follows: Blood from 20 mice was drawn by *Vena cava *puncture and platelet-poor plasma was obtained as described above. In rare cases the sampling technique could produce outliers. In order to exclude these artifacts from our negative control pool, each sample was tested for soluble fibrin and for thrombin-antithrombin complexes (method see below). The vast majority of samples were negative in these tests, and those samples were pooled. The pool was frozen in aliquots at -80°C.

### Measurement of soluble fibrin (SF) in mice

As a positive control, we incubated either purified human fibrinogen or human and murine plasma samples with 0.02 mU/ml bovine thrombin (Dade Behring, Liederbach, Germany). To prove the formation of crosslinked fibrin and fibrin-dimers, Western blotting of digested plasma or fibrinogen samples was performed. Samples were separated by SDS-PAGE (sodium dodecyl-sulfate polyacrylamide-gel electrophoresis) followed by capillary heat transfer onto nitrocellulose membranes. Membranes were stained with a polyclonal sheep-anti-rabbit fibrin(ogen) antibody (Haemochrom, Essen, Germany), known to be crossreactive with mouse and human fibrin(ogen), followed by chemiluminescent detection.

SF in plasma was then measured with two functional assays: i) a modification of a commercially available agglutination assay (FM-test, Roche-Diagnostics, Mannheim, Germany) which measures the agglutination reaction of SF with fibrin monomer-coated erythrocytes and ii) a modification of a chromogenic assay (Berichrom FM assay, Dade Behring), which is based on the ability of SF to enhance the tissue plasminogen activator (tPA)-induced plasminogen conversion. For the modified agglutination test, 5 μl of citrated mouse plasma were diluted in 100 μl of Owren's buffer (28 mM sodium barbital in 125 mM sodium chloride solution, pH = 7.35), and mixed on a slide (provided in the kit) with 50 μl of the erythrocyte suspension. After incubation at 37°C for 10 minutes, samples were mixed again and the agglutination reaction compared with positive and negative control samples. Positive controls were mouse plasma samples treated with 0.02 U/ml thrombin (Dade Behring), negative controls were untreated normal mouse plasma samples. For the chromogenic assay, 250 μl of human mini-plasminogen solution (provided in the kit) were pipetted into the wells of a 96 well microtiter plate, and the plate was preheated to 37°C. A standard curve was generated by incubating murine plasma samples with different amounts (2 mU/ml – 100 mU/ml) of bovine thrombin. 5μl samples and controls were added to the plasminogen-solution, and 50 μl tPA reagent was pipetted to each well with a multichannel pipettor. Samples were incubated at 37°C for 5 minutes, then 25 μl of plasmin substrate was added with a multichannel pipettor and absorption at 405 nm (minus 650 nm) was measured kinetically in an ELISA reader. The tests were also carried out with thrombin-treated or untreated human plasma.

To verify that the test results were due to the action of thrombin, we added the specific thrombin inhibitor hirudin. Hirudin (Roche, Mannheim, Germany) was either mixed *in vitro *at a concentration of 1.4 U/ml with the positive control samples, or injected in addition to LPS in the *in vivo *studies (see below).

### Other assays for coagulation parameters

For platelet and white blood cell counts, citrated blood was mixed with 1% (w/v) ammonium-oxalate (for platelet counts) or with 3% (v/v) acetic acid (for white blood cell counts) at a dilution of 1:20, and incubated on a shaker for 10 min. Platelets and white blood cells were then counted under a light microscope at 400 × magnification in a counting chamber (type Neubauer Improved). For schistocyte quantification, thin blood smears were prepared from full blood. Smears were dried and stained with the Pappenheim Method: Fixation in 100% (v/v) methanol, followed by 5 minutes staining in May-Grünwald solution (Sigma-Aldrich, St. Louis, MO) and 30 minutes in Giemsa solution (Sigma-Aldrich). Schistocytes were counted in three high power fields at 400 × magnification on a light microscope. Prothrombin time (PT) and activated partial thromboplastin time (aPTT) were automatically measured on a BCS coagulation analyzer (Dade Behring) using the Innovin kit (Dade Behring) for PT, the Actin FS kit (Dade Behring) for aPTT and normal human plasma (SHP, Dade Behring) as a standard. Thrombin-antithrombin complexes (TAT complexes) were measured using an ELISA test (Enzygnost-TAT, Dade Behring) according to the manufacturer's instructions, but using 20 μl of plasma samples and standards. This test is fully crossreactive with murine samples [13]. Briefly, microtiterplates which were coated with an anti-thrombin antibody were incubated with samples and standards (provided in the kit), washed, incubated with a peroxidase-conjugated anti-Antithrombin III antibody, washed and detected with OPD (o-phenylene-diamine-dihydrochloride) substrate. Absorption was measured on an ELISA reader. Normal platelet poor mouse plasma was used as a negative control. Antithrombin III (ATIII) was measured using a functional antithrombin test (Coamatic-Antithrombin, Chromogenix, Milano, Italy) according to the manufacturer's instructions, using 5 μl plasma and standards per test at the recommended dilutions. Briefly, plasma samples and standards (provided in the kit) were incubated with an excess of coagulation factor Xa in the presence of heparin. The residual factor Xa was quantified with a chromogenic substrate specific for coagulation factor Xa and the decrease of absorption was measured on an ELISA reader. Normal platelet poor mouse plasma was used as a standard to define 100% activity.

### Mouse models of systemic coagulation induction

Mice were injected either i.v. with 2 U bovine thrombin (Dade Behring) or i.p. with 20 μg or 50 μg lipopolysaccharides (LPS from *E.coli *serotype 055:B5, Sigma-Aldrich). For schistocyte counts, mice were injected with 20 μg LPS i.p. or with 100 μg tumor necrosis factor-alpha i.v. After specified time periods, citrated blood was drawn and analyzed for coagulation parameters as described above. In the cases where thrombin action was to be inhibited, 300 U hirudin were injected i.v. prior to 20 μg LPS.

### Tumor models

Immunodeficient mice were injected s.c. with 1 × 10^7 ^tumor cells. Tumor cell lines used were: F9 murine teratocarcinoma cells (ECACC, Salisbury, UK), Colo 677 human myeloma cells (DSMZ, Braunschweig, Germany), L540rec human Hodgkin's lymphoma cells [43], HT29 human colon carcinoma cells (ATCC/LGC, Middlesex, UK), LS174T human colon carcinoma cells (ATCC), IMR5 human neuroblastoma cells (kindly provided by Dr. Unwicker, University of Marburg, Germany). Mice were analyzed for coagulation parameters when tumors had reached a size of 150–500 μl. Differences between groups were analyzed for statistical significance with the Mann-Whitney U test for unpaired groups. The term "significant" was used, where p-values were less than 0.05.

### Coagulation parameters after treatment of tumor bearing mice with a VTA

Mice bearing Colo677 tumors were treated with a VTA developed in our laboratory. It consisted of a single chain variable fragment antibody (scFv) directed against murine vascular cellular adhesion molecule-1 (VCAM-1), which was expressed on tumor endothelial cells, and the extracellular domain of tissue factor. This fusion protein induces selective coagulation activation in the tumor vasculature [5]. One single dose of 20 μg was administered i.v. Mice were sacrificed at specified timepoints after treatment, and analysis of coagulation parameters was performed as described above.

## Competing interests

The author(s) declare that they have no competing interests.

## Authors' contributions

MU carried out the coagulation assays and animal studies, and participated in the drafting of the manuscript. AG participated in the design of the study and carried out the validation of the soluble fibrin test. CG conceived of the study and participated in its design, performance, coordination and helped to draft the manuscript. All authors have read and approved the final manuscript.
